# Outstanding Reviewers for *RSC Advances* in 2022

**DOI:** 10.1039/d3ra90053c

**Published:** 2023-06-26

## Abstract

We would like to take this opportunity to highlight the Outstanding Reviewers for *RSC Advances* in 2022, as selected by the editorial team for their significant contribution to the journal.
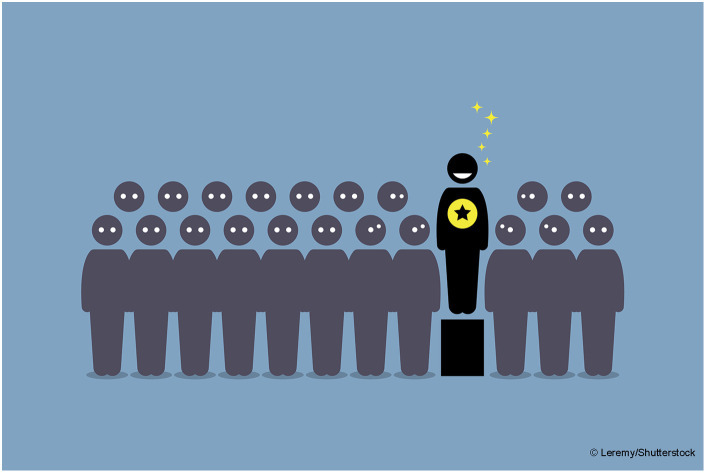

We would like to take this opportunity to thank all of *RSC Advances*' reviewers for helping to preserve quality and integrity in chemical science literature.

We would also like to highlight the Outstanding Reviewers for *RSC Advances* in 2022. Each one of our outstanding peer reviewers has been carefully selected by our editorial team and the list includes active researchers who have made significant contributions to peer review and have gone above and beyond in their actions.

In recognition of the varied contributions of our reviewer community, our Outstanding Reviewers from 2022 have been chosen based on several different measures, including the number, timeliness and quality of the reports completed over the last 12 months. We have also chosen to highlight reviewers who provided exceptionally thorough and detailed reports and reviewers who made additional efforts to aid authors in improving their manuscripts.

We would also like to direct a special thanks to the members of the *RSC Advances* Reviewer Panel for their hard work and dedication, and the valuable contribution they have made to the journal. The Reviewer Panel is a key part of our commitment to deliver rigorous and fair peer review and ensures that manuscripts are handled by experts throughout the peer review process. We are proud to work with these individuals and recognise their crucial role for the journal.

“*Peer review is a vital part of the publication process and relies upon the support of our authors and reviewers. We would like to take this opportunity to thank our Outstanding Reviewers and all our reviewers for their continued support for RSC Advances to allow us to maintain high publishing standards and fast decision times.*” – Karen Faulds, Editor-in-Chief

“*Our fantastic reviewer community provides the foundation on which the success of RSC Advances is built – I am really thankful to them for their commitment and expertise!*” – Russell J Cox, Editor-in-Chief

 

***RSC Advances* 2022 Outstanding Reviewers:**

 

Dr Takumi Abe

Okayama University

ORCID: 0000-0003-1729-1097

 

Dr Federico Bella

Politecnico di Torino

ORCID: 0000-0002-2282-9667

 

Dr Sambasiva R. Bheemireddy

Amionx, Inc.

ORCID: 0000-0003-1169-9649

 

Dr Shreyasi Chattopadhyay

University of St Andrews

ORCID: 0000-0003-4429-6117

 

Dr Marek Ingr

Tomas Bata University in Zlín

ORCID: 0000-0001-6741-9955

 

Dr Xiaochen Ji

Xiangtan University

ORCID: 0000-0001-9533-0376

 

Dr Pavan Kumar Chityala

BioMarin Pharmaceutical Inc.

ORCID: 0000-0003-3339-2920

 

Dr Maxim L. Kuznetsov

Instituto Superior Tecnico

ORCID: 0000-0001-5729-6189

 

Dr Jianbo Liu

Hunan University

ORCID: 0000-0001-8282-4078

 

Dr Masato Miyauchi

Japan Tobacco Inc., Tobacco Science Research Center

ORCID: 0000-0001-9005-9855

 

Dr Wenxuan Mo

South China University of Technology

ORCID: 0000-0001-9341-500X

 

Dr Dane Scott

Scott East Tennessee State University

ORCID: 0000-0003-0018-7189

 

Dr Harvijay Singh

Indian Institute of Technology Roorkee

ORCID: 0000-0002-8370-2037

 

Professor Carlos Torres-Torres

Instituto Politécnico Nacional

ORCID: 0000-0001-9255-2416

 

Dr Werner Ewald van Zyl

University of KwaZulu-Natal

ORCID: 0000-0002-2012-8584

 

Dr Anna S. Vikulina

Friedrich-Alexander-Universität Erlangen-Nürnberg

ORCID: 0000-0001-9427-2055

 

Dr Yunchao Xie

University of Missouri

ORCID: 0000-0001-6216-1211

 

Dr Zhi Yue

University of Chicago

ORCID: 0000-0002-4231-7474

 

Dr Li Zhang

Shanghai Second Polytechnic University

ORCID: 0000-0001-5774-4068

 

***RSC Advances* Reviewer Panel 2022 Outstanding Reviewers:**

 

Dr Sohini Bhattacharyya

Rice University

ORCID: 0000-0002-4626-1578

 

Dr Guillermo Bracamonte

National University of Cordoba

ORCID: 0000-0003-4760-3872

 

Dr Bin Chang

King Abdullah University of Science and Technology

ORCID: 0000-0003-4510-0550

 

Dr Lopamudra Das Ghosh

Texas A&M University

ORCID: 0000-0003-3867-6711

 

Dr S. Girish Kumar

RV College of Engineering, Department of Chemistry

ORCID: 0000-0001-9132-1202

 

Dr Darrick Heyd

Ryerson University

 

Dmitry Kharitonov

Jerzy Haber Institute of Catalysis and Surface Chemistry, Polish Academy of Sciences

ORCID: 0000-0003-2071-3975

 

Dr Gaurav Kumar

DuPont de Nemours Inc Water Solutions

ORCID: 0000-0001-7089-6146

 

Dr Shota Kuwahara

Toho University

ORCID: 0000-0001-7089-6146

 

Dr Hu Li

Guizhou University, Center for R&D of Fine Chemicals

ORCID: 0000-0003-3604-9271

 

Dr Jianmin Li

Zhejiang University

ORCID: 0000-0002-3917-8653

 

Dr Feng Li

The University of Sydney

ORCID:0000-0003-4448-074X

 

Dr Guangchao Liang

Xidian University

ORCID: 0000-0001-7235-958X

 

Dr Ekkenhard Lindner

Institut für Anorganische Chemie, Universität Tübingen

 

Dr Lingaiah Maram

University of Health Sciences and Pharmacy in St Louis

ORCID: 0000-0003-1327-8426

 

Professor Angel Meléndez

Universidad Industrial de Santander

ORCID: 0000-0002-5166-1840

 

Dr Wenli Pei

Northeastern University

ORCID: 0000-0003-2525-152X

 

Dr Abhispa Sahu

American Nano LLC

ORCID: 0000-0002-3223-7577

 

Dr Paresh Kumar Samantaray

Chemistry and Chemical Engineering, California Institute of Technology

ORCID: 0000-0003-2533-929X

 

Professor Beatriz Sánchez

Universidad de Alcala de Henares

ORCID: 0000-0002-6584-1949

 

Dr James Sheehan

The University of Alabama

ORCID: 0000-0001-5548-8099

 

We would also like to thank the *RSC Advances* Editorial Board and Associate Editors and the chemistry community for their continued support of the journal, as authors, reviewers and readers.

We continue to work on improving the diversity of our reviewer pool to reflect the diversity of the communities that we serve.

 

Russell Cox, Editor-in-Chief

Karen Faulds, Editor-in-Chief

Laura Fisher, Executive Editor

## Supplementary Material

